# Human Cytomegalovirus Protein pUL117 Targets the Mini-Chromosome Maintenance Complex and Suppresses Cellular DNA Synthesis

**DOI:** 10.1371/journal.ppat.1000814

**Published:** 2010-03-19

**Authors:** Zhikang Qian, Van Leung-Pineda, Baoqin Xuan, Helen Piwnica-Worms, Dong Yu

**Affiliations:** 1 Department of Molecular Microbiology, Washington University School of Medicine, Saint Louis, Missouri, United States of America; 2 Department of Cell Biology and Physiology, Washington University School of Medicine, Saint Louis, Missouri, United States of America; 3 Department of Internal Medicine, Washington University School of Medicine, Saint Louis, Missouri, United States of America; 4 Howard Hughes Medical Institute, Washington University School of Medicine, Saint Louis, Missouri, United States of America; Oregon Health and Science University, United States of America

## Abstract

Modulation of host DNA synthesis is essential for many viruses to establish productive infections and contributes to viral diseases. Human cytomegalovirus (HCMV), a large DNA virus, blocks host DNA synthesis and deregulates cell cycle progression. We report that pUL117, a viral protein that we recently identified, is required for HCMV to block host DNA synthesis. Mutant viruses in which pUL117 was disrupted, either by frame-shift mutation or by a protein destabilization-based approach, failed to block host DNA synthesis at times after 24 hours post infection in human foreskin fibroblasts. Furthermore, pUL117-deficient virus stimulated quiescent fibroblasts to enter S-phase, demonstrating the intrinsic ability of HCMV to promote host DNA synthesis, which was suppressed by pUL117. We examined key proteins known to be involved in inhibition of host DNA synthesis in HCMV infection, and found that many were unlikely involved in the inhibitory activity of pUL117, including geminin, cyclin A, and viral protein IE2, based on their expression patterns. However, the ability of HCMV to delay the accumulation of the mini-chromosome maintenance (MCM) complex proteins, represented by MCM2 and MCM4, and prevent their loading onto chromatin, was compromised in the absence of pUL117. When expressed alone, pUL117 slowed cell proliferation, delayed DNA synthesis, and inhibited MCM accumulation. Knockdown of MCM proteins by siRNA restored the ability of pUL117-deficient virus to block cellular DNA synthesis. Thus, targeting MCM complex is one mechanism pUL117 employs to help block cellular DNA synthesis during HCMV infection. Our finding substantiates an emerging picture that deregulation of MCM is a conserved strategy for many viruses to prevent host DNA synthesis and helps to elucidate the complex strategy used by a large DNA virus to modulate cellular processes to promote infection and pathogenesis.

## Author Summary

Inhibition of host DNA synthesis is pivotal for many viruses to establish productive infection and cause disease. Human cytomegalovirus (HCMV) is the top viral cause of birth defects in newborns and leads to life-threatening diseases in individuals with compromised immunity. HCMV blocks host DNA synthesis and creates a cellular environment to replicate its own genome. We report here that pUL117, a novel viral protein that we recently identified, is required for HCMV to block host DNA synthesis. Mechanistically, pUL117 is necessary and sufficient to reduce the accumulation of the mini-chromosome maintenance (MCM) complex, a replicative helicase that unwinds the origin and initiates cellular DNA replication. During HCMV infection pUL117 may also have a direct role in preventing MCM loading onto chromatin. Importantly, knockdown of MCM proteins restored the ability of pUL117-deficient virus to block cellular DNA synthesis. Thus, targeting MCM function is a mechanism for pUL117 to help block cellular DNA synthesis during HCMV infection. Several proteins encoded by other viruses have also been reported to subvert MCM function by distinct mechanisms and inhibit host DNA synthesis when over-expressed in host cells. Therefore, MCM has emerged as a conserved target for viruses to prevent host DNA synthesis. Our results illustrate a novel strategy that HCMV uses to manipulate this critical cellular factor during infection. This study helps to elucidate the sophisticated strategies used by a large DNA virus to modulate cellular processes to promote infection and pathogenesis and may also shed light on the regulation of eukaryotic DNA replication.

## Introduction

The manipulation of host DNA synthesis is a critical step for many DNA viruses, including human cytomegalovirus (HCMV), to establish productive infection leading to disease [1],[2],[3]. HCMV is a prototypical β-herpesvirus, a ubiquitous pathogen, and one of the most common causes of birth defects in newborns and life-threatening disease in immunocompromised individuals. Despite its slow replication kinetics, HCMV can efficiently infect, persist, and establish latency in humans. To accomplish this, HCMV encodes at least 166 annotated genes, many of which act to hijack and deregulate key cellular processes, such as cellular DNA synthesis and cell cycle control [4].

HCMV infection induces a cellular environment conducive for DNA replication but in the meantime specifically blocks host DNA synthesis, thereby arresting host cells in a pseudo-G1 phase [5],[6]. It is proposed that such modulation allows the virus to divert resources, such as energy, nucleotide pools, and cellular DNA replication enzymes, exclusively for viral DNA replication. Consistent with this notion, cells that actively replicate their DNA fail to support a lytic HCMV infection. Instead, they progress through S phase and arrest at the next G1-phase after mitosis to initiate HCMV replication [7],[8].

HCMV encodes multiple factors, both stimulatory and inhibitory, to tightly regulate host and viral DNA replication for a successful infection (reviewed in [5],[6],[9]). Over-expression of HCMV protein pp71, IE1, IE2, or pUL97 inactivates pRb-family proteins, activates expression of E2F-dependent S-phase genes, and promotes G1/S- transition. On the other hand, pUL69 [10],[11] and IE2 [12] are required for HCMV to block cellular DNA synthesis. Over-expression of IE2 inhibits cyclin A transcription, induces p16 expression, and arrests cells in S phase [13],[14],[15],[16]. The mechanism for the cell-cycle arresting activity of pUL69 remains unknown. These viral factors must act in conjunction at multiple regulatory levels in order for HCMV to manipulate the host cell cycle. Neither IE2 nor pUL69 alone blocks cellular DNA synthesis during infection, because a mutant HCMV expressing one but not the other fails to arrest host cells at a pseudo-G1 phase [10],[12]. In addition to cellular proteins involved in cell cycle control, HCMV directly targets DNA replication factors, such as mini-chromosome maintenance (MCM) complex [17],[18]. MCM, a hetero-hexameric protein complex composed of MCM2-MCM7, is recruited by Cdc6 and Cdt1 to replication origins, forming pre-replication complexes (pre-RC) during late M- to G1- phase to allow replication licensing. Once activated by cyclin-dependent kinase 2 and Dbf4-dependent kinase (Dbf4/cdc7) in S phase, MCM acts as a replicative helicase to unwind the origin and initiate DNA replication [19]. HCMV inhibits loading of MCM onto chromatin to prevent cellular replication licensing [17],[18], and this inhibitory activity appears to correlate with virus-induced early accumulation of geminin, a negative regulator of the assembly of pre-replication complexes [18].

We previously identified a protein product of HCMV early gene UL117, termed pUL117 [20]. We showed that pUL117-deficient HCMV replicated viral DNA at slightly reduced levels but was markedly delayed in the maturation of viral replication compartments and severely attenuated in growth in human foreskin fibroblasts [20]. In this study, we investigated the potential role of pUL117 in manipulating host cells during HCMV infection. We report that pUL117 is necessary and sufficient to reduce the accumulation of MCM proteins. During HCMV infection pUL117 may also have a direct role in preventing MCM loading onto chromatin. Importantly, knockdown of MCM proteins restored the ability of pUL117-deficient virus to block cellular DNA synthesis. Thus, targeting MCM function is a mechanism for pUL117 to help block cellular DNA synthesis during HCMV infection. These findings reveal a novel strategy used by HCMV to usurp the host DNA replication machinery, and provide new insight into the complex host-pathogen interactions fundamental to the biology and pathogenesis of the virus.

## Results

### pUL117 is required for HCMV to block cellular DNA synthesis during infection

**Figure 1:**
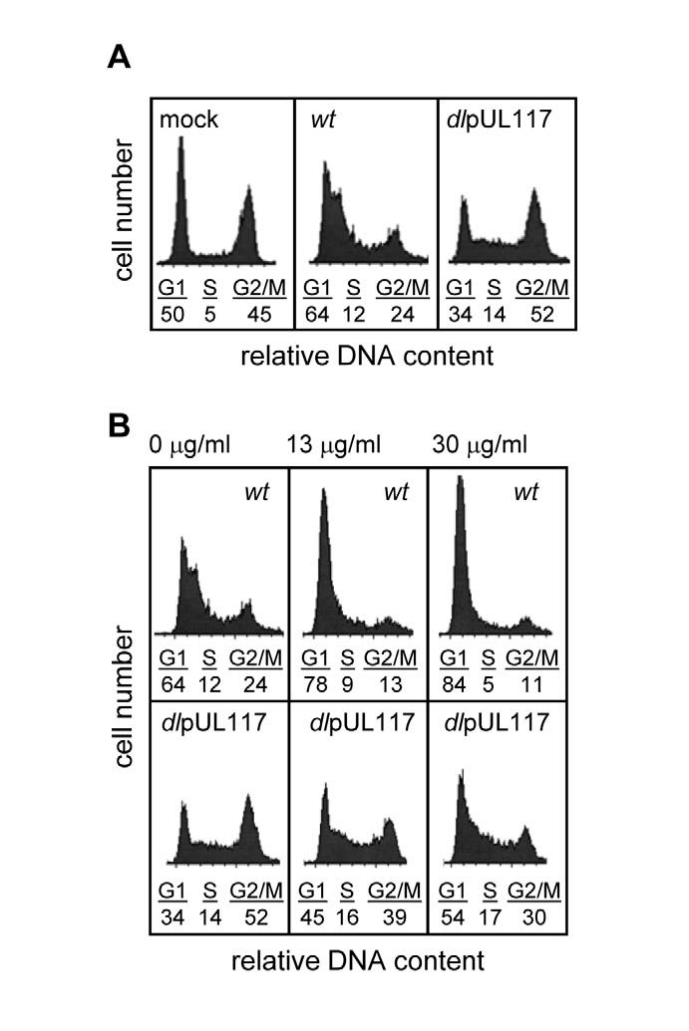
UL117-deficient virus failed to block host DNA synthesis during infection of actively growing human foreskin fibroblasts (HFFs). Actively growing HFFs were mock-infected, or infected with wild type HCMV (*wt*), or pUL117-deficient virus (*dl*pUL117), and nocodozale was added 8 hours post infection (hpi). At 48 hpi, cells were stained with propidium iodide (PI) and their DNA content was determined by flow cytometry based cell cycle analysis. (A) HFFs that were mock-infected or infected with recombinant HCMV without gancyclovir (GCV) treatment. (B) HFFs that were infected in the presence of GCV at indicated concentrations.

We previously discovered that pUL117 was required for the proper maturation of nuclear viral replication compartments and efficient HCMV infection in primary human foreskin fibroblasts (HFFs) [20]. Here, we asked if pUL117 functioned by targeting host factors involved in cellular DNA replication and cell cycle control. HCMV infection blocks cellular DNA synthesis and predominantly arrests permissive cells in a phenotypically G1-like phase [5],[6]. To test whether this activity required pUL117, actively growing HFFs were mock-infected, infected with wild type virus (AD*wt*) or pUL117-deficient virus (AD*dl*pUL117). Nocodazole was added 8 hours post infection (hpi) to prevent cell division, and the DNA content was analyzed by flow cytometry-based cell cycle analysis at 48 hpi (Fig. 1A). 45% of mock-infected cells reached G2/M by this time point. As anticipated, wild type HCMV infection efficiently blocked host DNA synthesis with only 24% of cells reaching G2/M at 48 hpi, and this population of cells likely represented uninfected bystanders or abortively-infected cells. However, pUL117-deficient virus failed to block host DNA synthesis, allowing 52% of cells to progress to G2/M. In wild type virus infected cells, broadening of the G1 peak was observed as a result of viral DNA synthesis [21]. Broadening of the G1 peak was less evident in AD*dl*pUL117 infected cells at this time point, likely due to the slight delay of viral DNA synthesis [20]. To eliminate potential complications due to viral DNA synthesis, we also analyzed the DNA content of cells after infection in the presence of ganciclovir (GCV), a specific inhibitor of HCMV DNA synthesis (Fig. 1B). The results showed that GCV, at concentrations of 13 and 30 µg/ml, blocked the broadening of the G1 peak, indicative of viral DNA synthesis inhibition. Thus, while wild type HCMV maintained its ability to block host DNA synthesis, mutant virus failed to do so and cells entered S phase and progressed to G2/M phase.

**Figure 2:**
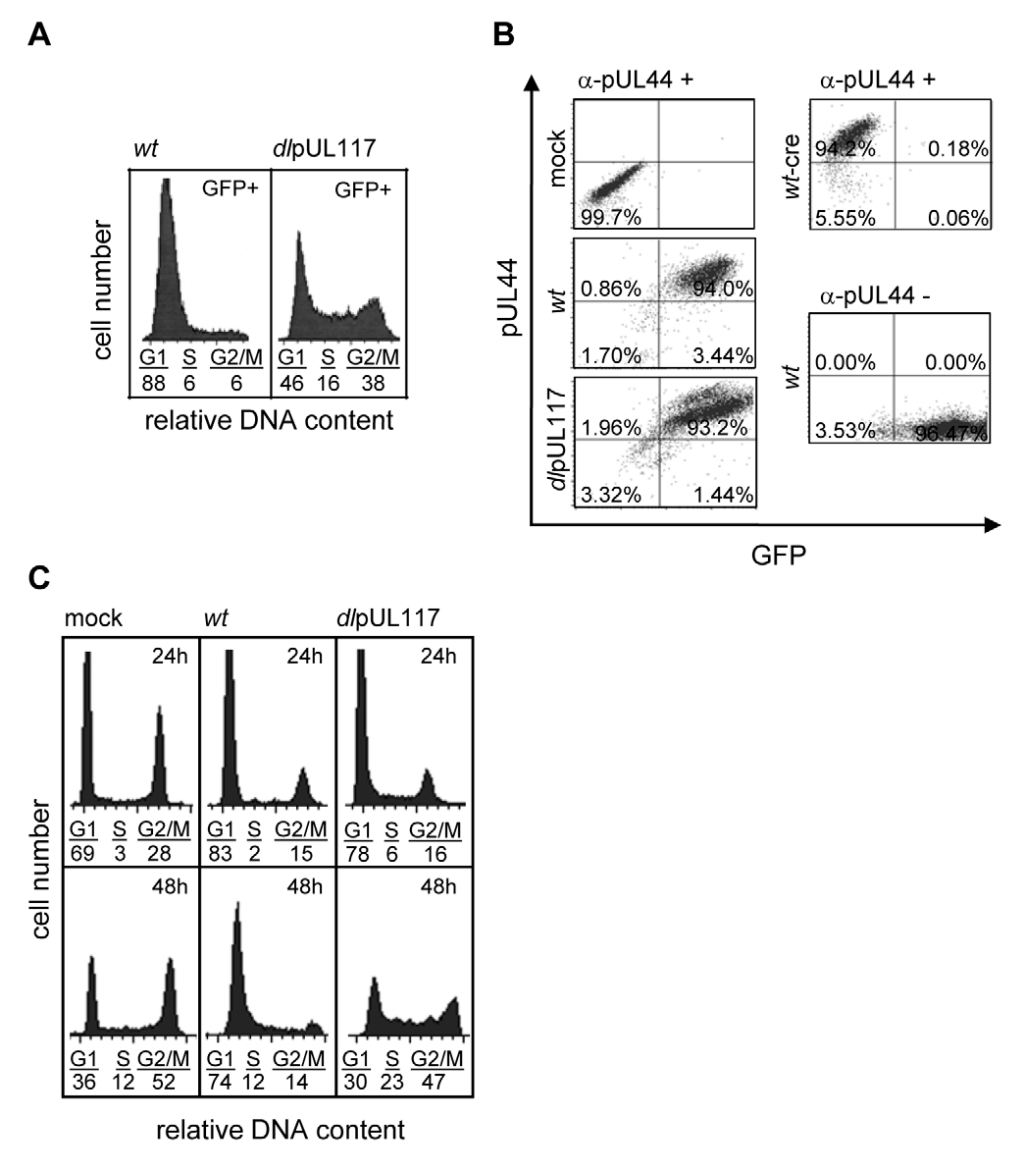
pUL117 was required to block host DNA synthesis of HCMV-infected cells at late times during infection. Actively growing HFFs were mock-infected, or infected with wild type HCMV (AD*wt*), or pUL117-deficient virus (AD*dl*pUL117), and nocodozale was added 8 hpi. (A) Cells were sorted by HCMV-driven GFP expression at 48 hpi, stained with PI, and GFP+ cells were analyzed for their DNA content by flow cytometry. (B) Cells were fixed and labeled with the antibody to viral protein pUL44 (α-pUL44) at 48 hpi. Cellular distributions of GFP and pUL44 signals were analyzed by two-color flow cytometry. Specificity of GFP or pUL44 signal was demonstrated by analyzing infection of recombinant HCMV AD*wt-cre* that did not express GFP [53] or AD*wt* without pUL44 staining, respectively. (C) Cells were infected in the presence of GCV (30µg/ml), double-stained with PI and the antibody to viral proteins IE1/IE2 at 24 and 48 hpi, and IE1/IE2+ cells were determined for their DNA content.

As all HCMV viruses used in this study were tagged with GFP, we specifically analyzed the DNA content of GFP-positive infected cells by sorting cells based on GFP expression. Sorting was efficient as 99% of sorted cells were GFP positive (data not shown). Upon AD*wt* infection, only 6% of infected cells reached the S- or G2/M- phase, whereas upon AD*dl*pUL117 infection, 16% and 38% of cells reached S and G2/M, respectively (Fig. 2A). Thus, unlike AD*wt* infection, the mutant virus did not block host DNA synthesis. It is known that abortive HCMV infection is unable to block host DNA synthesis [22],[23],[24]. However, this was not the case here because pUL117-deficient virus established productive infection, producing infectious progeny even though the kinetics of its viral DNA replication was slightly delayed in HFFs [20]. This was further supported by the observation that 93–94% of wild type and mutant virus infected cells that were GFP-positive also expressed early viral protein pUL44, confirming that both viruses were able to establish productive infection equally well (Fig. 2B and Figure S4). In this study, we used HCMV-driven GFP or viral proteins pUL44 (DNA polymerase processivity factor) or IE1/IE2 (immediate early proteins) interchangeably as markers of productive infection as they all identified the same cell populations during infection (data not shown).

To examine the effect of pUL117 on host DNA synthesis more closely during infection, we gated infected cells by IE1/IE2 and analyzed their DNA content at both 24 and 48 hpi (Fig. 2C). Infection was carried out in the presence of GCV to prevent viral DNA replication. At 24 hpi, although slightly more mutant virus-infected cells (6% and 16%) than wild type virus-infected cells (2% and 15%) progressed to S-phase and G2/M phase, the mutant infection still blocked host DNA synthesis quite efficiently when compared to mock infection (3% and 28%) (Fig. 2C). However, as expected, while AD*wt* infection maintained this inhibitory activity at 48 hpi, the mutant virus was unable to block host DNA synthesis of actively growing HFFs at this time point.

**Figure 3:**
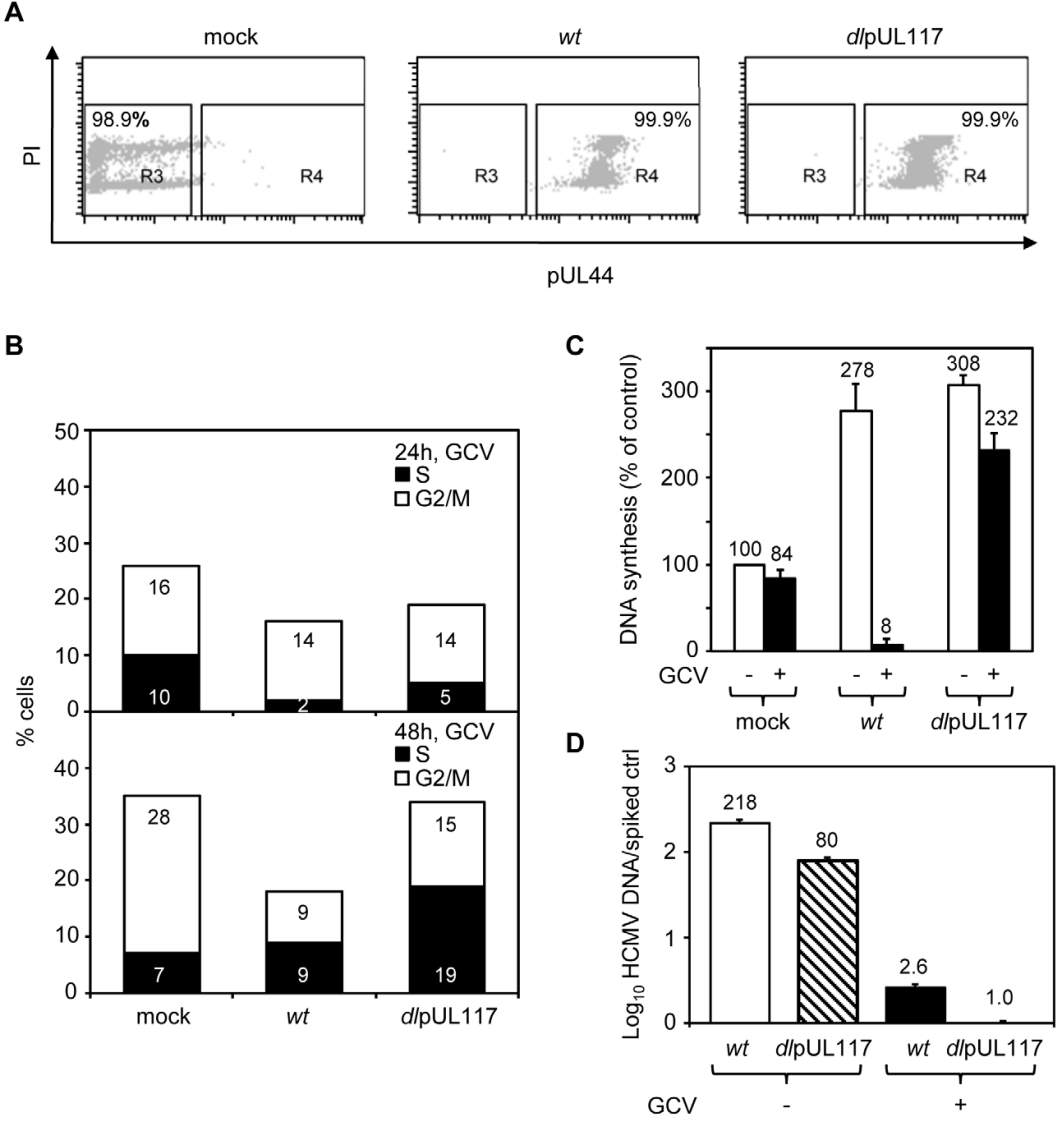
pUL117 was required to block host DNA synthesis of G0-synchronized HFFs during HCMV infection. Subconfluent HFFs were synchronized at G0 by serum starvation and then infected with recombinant HCMV in the presence of serum. Nocodazole was added at 8 hpi. (A and B) Cells were infected in the presence of GCV (30µg/ml), double-stained with PI and α-pUL44 at 24 and 48 hpi, and analyzed by flow cytometry for their DNA content and pUL44 expression. Shown is a representative of three reproducible, independent experiments. (A) Signal profiles of pUL44 (x-axis) and PI (y-axis) of mock- or virus- infected cells at 48 hpi. Cells within gate R3 or R4 represents pUL44-negative or pUL44-positive cells, respectively. (B) Mock-infected cells in R3 and virus-infected cells in R4 were analyzed for their DNA content. Percentages of cells in S-phase or G2/M-phases were shown as solid or open bars, respectively. (C) The rate of DNA synthesis of infected cells with or without GCV (30µg/ml) was measured by [^3^H] thymidine incorporation at 48 hpi. Shown are relative amounts of incorporated [^3^H] thymidine in each sample with thymidine incorporation in mock-infected cells in the absence of GCV set at 100%. (D) Accumulation of viral DNA in infected cells with or without GCV (30µg/ml) was measured by realtime-quantitative PCR at 48 hpi.

To better define how pUL117 modulates host DNA synthesis, we examined its impact on the DNA profile of HFFs that were synchronized at G0 prior to HCMV infection, a condition that has been widely used to investigate the modulation of cell cycle and cellular DNA synthesis by HCMV. We synchronized subconfluent HFFs at G0 by serum starvation, infected them with mock or recombinant HCMV in the presence of serum, GCV, and nocodazole, and analyzed the DNA content of infected cells that were gated for expression of viral protein pUL44 (Fig. 3). More than 99% of cells infected with either wild type or mutant virus were pUL44 positive, indicating a robust productive infection (Fig. 3A). The overall cell cycle progression of serum-stimulated quiescent mock-infected cells or cells infected with mutant virus was delayed compared to actively growing cells. Nonetheless, as anticipated, while a total of 35% of mock-infected cells moved into S-phase (7%) and G2/M-phase (28%) at 48 hpi, wild type HCMV blocked host DNA synthesis, resulting in only 18% of pUL44+ cells moving into S-phase (9%) and G2/M-phase (9%). In contrast, pUL117-deficient virus failed to block host DNA synthesis, and 34% of pUL44+ cells moved into S-phase (19%) and G2/M-phase (15%) (Fig. 3B). This defect was further confirmed by direct measurement of DNA synthesis with ^3^H thymidine incorporation at 48 hpi (Fig. 3C). The amount of GCV-resistant, replicating DNA in cells infected with mutant virus (232%) was ∼30-fold higher than that in cells infected with wild type virus (8%).

The resistance of DNA replication in pUL117 deficient virus-infected cells to GCV indicated that we were selectively monitoring cellular DNA synthesis. In support of this, GCV was found to inhibit viral DNA synthesis equally well in cells infected with wild type or mutant virus (Fig. 3D). GCV treatment resulted in ∼80-fold reduction of viral DNA accumulation in both wild type and mutant virus infected cells at 48 hpi. Importantly, in the presence of GCV, the residual viral DNA in mutant virus infected cells was 2.6-fold less than that in wild type virus infected cells. Furthermore, the elevated DNA content seen in cells infected with mutant virus as compared to wild type virus was recapitulated when viral DNA replication was inhibited by another specific viral DNA synthesis inhibitor, phosphonoacetic acid (PAA) (Figure S1).

It was noted that, similar to infection in actively growing HFFs (Fig. 2C), in G0-synchronized HFFs the inability of pUL117-deficient virus to block host DNA synthesis at 24 hpi was less pronounced than that at 48 hpi (Fig. 3B and Figure S1). It is also important to note that progress through S-phase was delayed during infection of mutant virus as compared to mock-infected cells at 48 hpi, both in actively growing HFFs (Fig. 2C, 23% of infected cells versus 12% of mock) and in G0-synchronized HFFs (Fig. 3B, 19% of infected cells versus 7% of mock). These results suggest that other viral factors, such as IE2 and pUL69, are able to block host DNA synthesis at early times and they may also institute additional blockades in S phase in the absence of pUL117 at times after 24 hpi.

Collectively, these results are consistent with the notion that HCMV encodes multiple factors to modulate the DNA replication machinery of host cells, and importantly, our results demonstrate that pUL117 is required for HCMV to block host DNA synthesis at times after 24 hpi.

### HCMV has an ability to stimulate cellular DNA synthesis in the absence of pUL117

**Figure 4:**
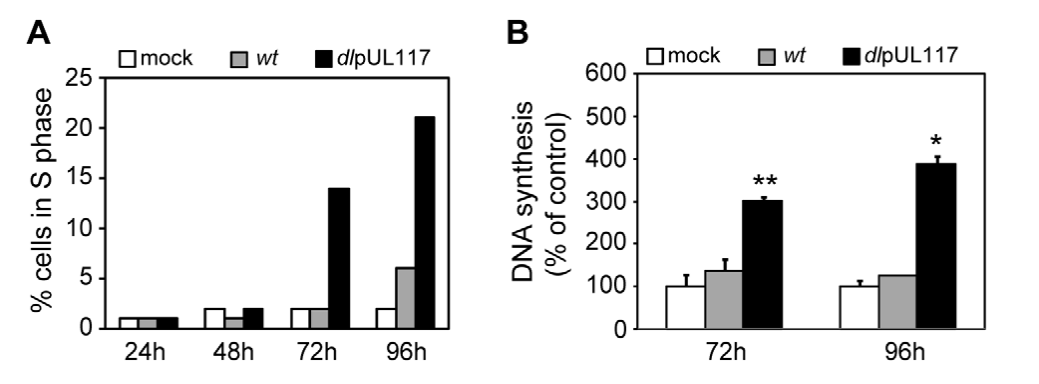
pUL117-deficient virus stimulated host DNA synthesis of quiescent HFFs in the absence of serum. HFFs were synchronized at G0 by serum starvation and contact inhibition, and then infected with recombinant HCMV with GCV (30µg/ml) but without serum. (A) Nocodazole was added at 8 hpi and the percentage of IE1/IE2+ cells in S-phase was determined by flow cytometry at indicated times post infection. Shown is a representative of three reproducible, independent experiments. (B) The rate of DNA synthesis of infected HFFs at 72 or 96 hpi was measured by [^3^H] thymidine incorporation. *, *P*<0.05; **, *P*<0.01 by Student's *t* test.

A major strategy employed by many DNA viruses to replicate their genomes is to promote host cell entry into S-phase in order to utilize the cellular resources needed for viral DNA synthesis. The HCMV UL97 protein has cyclin-dependent kinase (CDK) activity, allowing the virus to inactivate Rb-family proteins and activate transcription of S-phase genes [1],[25]. Earlier studies have also shown that HCMV induces the expression of cellular genes needed for DNA replication, such as the ones encoding the pre-RC components [17],[18]. Thus, HCMV may have an “intrinsic” ability to stimulate cellular DNA synthesis, and that this stimulatory activity is counteracted by pUL117. This hypothesis was supported by the observation that when infecting cycling cells, AD*dl*pUL117 infection promoted a greater number of cells to move out of G1 compared to mock-infected cells at 48 hpi (Fig. 2C) and 72 hpi (data not shown). To test the hypothesis directly, we synchronized HFFs in G0 by contact inhibition and serum starvation, infected them with HCMV in the presence of GCV and nocodazole but without serum (to prevent serum-stimulated re-entry of cells into the cell cycle), and then analyzed infected cells for their DNA content by flow cytometry and for DNA synthesis by ^3^H thymidine incorporation (Fig. 4). At 96 hpi, while very few mock infected (2%) or wild type virus-infected cells (6%) entered S phase, pUL117-deficient virus stimulated entry of host cells into S phase (21%) (Fig. 4A). Direct measurement of thymidine incorporation confirmed elevated levels of DNA replication in mutant virus-infected cells under conditions of serum starvation and contact inhibition (Fig. 4B). This result suggests that HCMV has the intrinsic ability to drive host cells to initiate cellular DNA synthesis, but this stimulatory activity is blocked by pUL117.

### Inability of pUL117-deficient virus to block host DNA synthesis is due to the absence of pUL117 expression

It was conceivable that viral stocks of AD*dl*pUL117 used for infection in our study might differ from those of wild type virus in composition or virion structures, and this physical difference of the seed stocks rather than the lack of pUL117 expression during infection would directly alter the modulation of cellular DNA synthesis when cells were infected. This was unlikely since there was no evidence for the presence of pUL117 in HCMV virions [26] and the mutant virus was capable of blocking cellular DNA synthesis at early times during infection (Figs. 2C and 3A). Nonetheless, to provide direct evidence we made a recombinant HCMV in which the UL117 ORF was tagged with a FKBP destabilization domain (ddFKBP) at its amino terminus (ADpFKBP-UL117). The fusion protein pFKBP-UL117 was directed for rapid degradation by ddFKBP, but could be stabilized by the ligand Shield-1 (Shld1) that binds to ddFKBP [27],[28]. This approach has recently been used to generate conditional HCMV mutants in which ddFKBP was fused to three essential viral genes to regulate accumulation of these fusion proteins and viral growth [29]. This approach provided a rapid and reversible way to regulate the accumulation of pUL117 in infections from the same viral stock. We predicted that infection would be pUL117-deficient without Shld1 (pFKBP-UL117 unstable) but would be wild type with Shld1 (pFKBP-UL117 stable).

**Figure 5:**
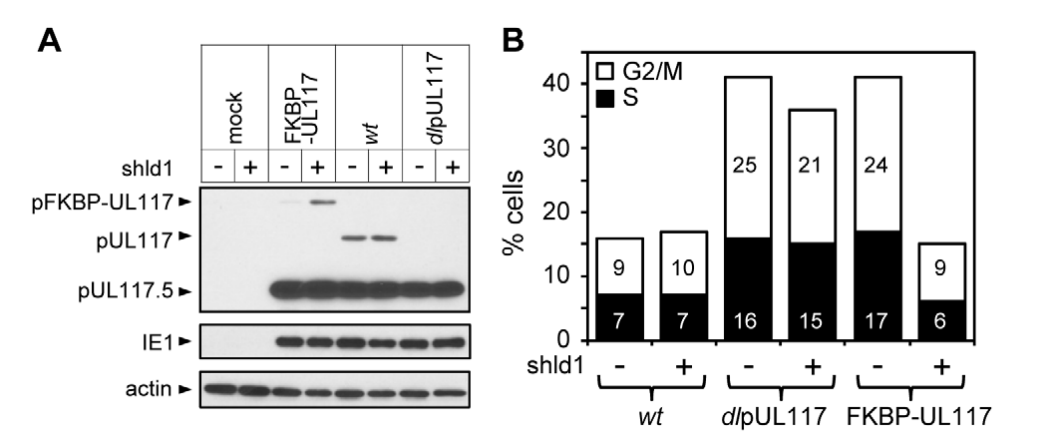
Lack of newly synthesized pUL117 was directly responsible for the failure of pUL117-deficient virus to block host DNA synthesis. Recombinant HCMV virus ADpFKBP-UL117 carried a functional pUL117 variant that was tagged with a FKBP destabilization domain at its N terminus (pFKBP-UL117). HFFs were infected with ADpFKBP-UL117 with or without stabilizing ligand Shld1 (1µM). Nocodozale was added at 8 hpi, infected cells were harvested at 48 hpi, the accumulation of pUL117 or pFKBP-UL117 was determined by immunoblotting analysis (A), and the DNA content by flow cytometry (B). Percentages of cells in S-phase or G2/M-phases were shown as solid or open bars, respectively.

HCMV virus prepared without Shld1 was used to infect actively growing HFFs incubated with or without Shld1, and viral protein accumulation and cell cycle distribution of infected cells were analyzed at 48 hpi. Shld1 only had negligible effect on accumulation of viral proteins pUL117, pUL117.5, and IE1 (Fig. 5A), modulation of the cell cycle progression (Fig. 5B), or replication of AD*wt* or AD*dl*pUL117 (data not shown). With Shld1, the pFKBP-UL117 fusion protein was stabilized and ADpFKBP-UL117 effectively blocked host DNA synthesis. In contrast, without Shld1, the accumulation of the FKBP-UL117 fusion protein was greatly reduced (Fig. 5A) and recombinant virus failed to prevent host DNA synthesis (Fig. 5B). Thus, altered modulation of host DNA synthesis in pUL117-deficient virus was the direct result of abrogation of pUL117 expression rather than physical differences between wild type and mutant viral stocks.

### pUL117 blocks accumulation of MCM proteins and their loading onto chromatin during HCMV infection

**Figure 6:**
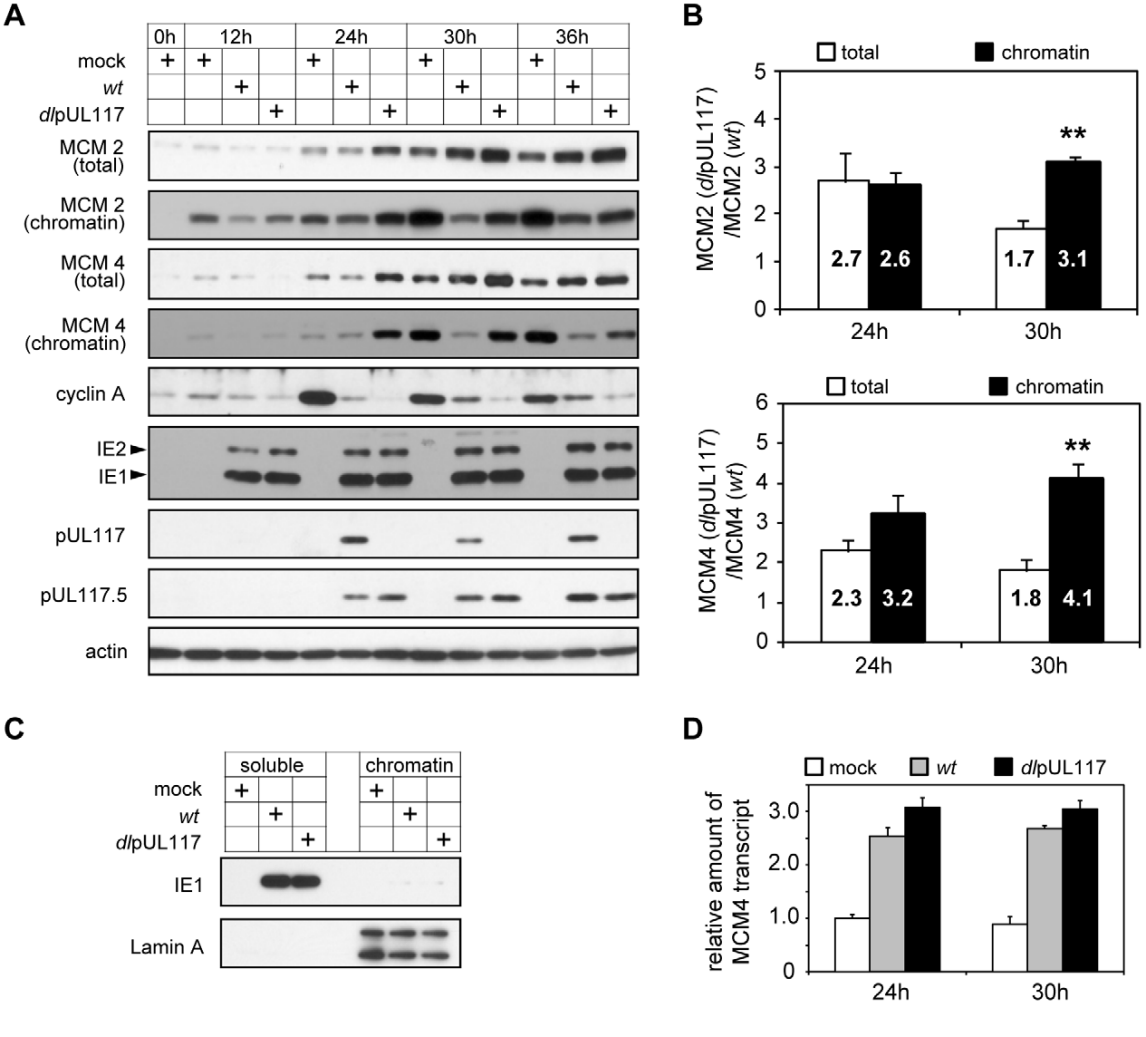
pUL117-deficient virus failed to block accumulation and chromatin loading of MCM components. Subconfluent HFFs were synchronized at G0 by serum starvation, and then infected with recombinant HCMV in the presence of serum. (A) At indicated times, total lysate or chromatin fractions from equal number of cells were analyzed by immunoblotting for viral and cellular factors known to modulate the cell cycle and DNA replication. Also shown were pUL117-related proteins and actin control. (B) Amounts of MCM2 and MCM4 in total cell lysates or chromatin fractions from equal number of infected cells at 24 and 30 hpi were quantified as described in Materials and Methods. The ratio of MCM in mutant virus infection to that in wild type virus infection was calculated. Shown are the averages of the results from three independent sets of infections. *, *P*<0.05; **, *P*<0.01 by Student's *t* test. (C) The completion of fractionation was determined by immunoblotting of viral protein IE1 and cellular protein Lamin A in soluble and chromatin fractions. (D) At indicated times post infection, RNA from infected cells was extracted, treated with TURBO DNA-free reagent (Ambion) to remove contaminating DNA, and the amounts of MCM4 and GAPDH control transcripts were determined by reverse transcription-couple realtime-quantitative PCR analysis. All data sets were within the linear ranges of standard curves shown in Figure S5. The amount of MCM4 transcript was then normalized to that of GAPDH transcript. For each time point, the normalized amount of MCM4 transcript in mock infection was set at 1.

To dissect the mechanism of pUL117 function, we examined several key cellular factors that regulate cellular DNA replication and are perturbed by HCMV infection. We were particularly interested in events occurring at times prior to the failure of the pUL117-deficient virus to block host DNA synthesis (i.e., 0–36 hpi) as misregulation of these events was the most likely cause of the defect. HCMV up-regulates p16 at late times [13],[30], inhibits cyclin A accumulation [8],[31],[32], and prevents MCM complex loading onto chromatin [17],[18]. When infecting G0-synchronized HFFs in the presence of serum, HCMV induced p16 accumulation and expressed another viral inhibitory protein IE2 independent of pUL117 (Fig. 6A and Figure S2). HCMV also regulated the accumulation of two other cellular regulatory proteins, p53 [31] and p21 [32], independent of pUL117 (Figure S2). Both wild type and pUL117-deficient viruses inhibited accumulation of cyclin A during infection (Fig. 6A). This was anticipated because the mutant virus expressed IE2, which has been known to inhibit cyclin A accumulation [14], and was consistent with S-phase delay observed in mutant virus infection (Figs. 2C and 3A). Interestingly, mutant virus appeared to inhibit cyclin A to an even greater extent than wild type virus (Fig. 6A). This inhibition could not explain elevated cellular DNA seen in mutant virus infection because in normal cells cellular DNA synthesis and S-phase progression correlates with cyclin A accumulation. This result also suggested that the cell cycle proceeded, albeit with delayed kinetics, in fibroblasts even when cyclin A level was reduced. Indeed, it has been shown that depletion of cyclin A does not prevent proliferation of fibroblasts [33] or cellular DNA synthesis [34]. Thus, we found no evidence for a role of viral protein IE2, cellular cell cycle regulatory proteins p16, p21, p53, or cyclin A in elevated cellular DNA synthesis in pUL117-deficient virus infection.

In striking contrast, the ability of HCMV to modulate the MCM complex, a key component of replication licensing, was altered in the absence of pUL117 (Fig. 6A). In mock-infected cells, MCM proteins, represented by MCM2 and MCM4, were almost undetectable at G0 (0 h) but were expressed and recruited to chromatin when cells re-entered the cell cycle (12–36 h). As reported previously [17],[18], HCMV infection slightly reduced MCM accumulation at early times (12–24 h) but induced their overall levels at late times (30–36 h), and MCM accumulation in chromatin fractions from wild type HCMV-infected cells was markedly reduced. The purity of our fractions was confirmed by demonstrating that cellular protein lamin A was exclusively present in chromatin fractions but not in soluble fractions, and the vast majority of viral protein IE1 was present in soluble fractions but not in chromatin fractions (Fig. 6C). pUL117 mutant virus infection induced even higher MCM accumulation and failed to efficiently block MCM recruitment to chromatin as compared to wild type virus infection (most evident at 24–30 hpi). This difference between wild type and mutant virus infection was apparent for both MCM2 and MCM4, although the effect on MCM4 was greater. The altered accumulation of MCM proteins was likely to occur at a post-transcriptional level as MCM4 transcript accumulated at similar levels during HCMV infection independent of pUL117 (Fig. 6D).

To better define the effect of pUL117 on MCM accumulation and chromatin loading, we quantified total and chromatin bound MCM protein levels at 24 and 30 hpi. It was clear that both total and chromatin bound MCM levels markedly increased in mutant virus infection relative to wild type virus infection (Fig. 6B). At 24 hpi, the increase of chromatin-bound MCM protein levels paralleled the increase of total MCM levels in mutant virus infection. However, at 30 hpi, chromatin bound MCM2 and MCM4 increased by 3.2- and 4.1- fold whereas total MCM2 and MCM4 only increased by 1.7- and 1.8- fold, respectively, in the absence of pUL117. These results suggest that pUL117 inhibits replication licensing by reducing accumulation of total MCMs (e.g. 24 hpi), and additionally, may by targeting MCM chromatin loading (e.g. 30 hpi) during HCMV infection.

**Figure 7:**
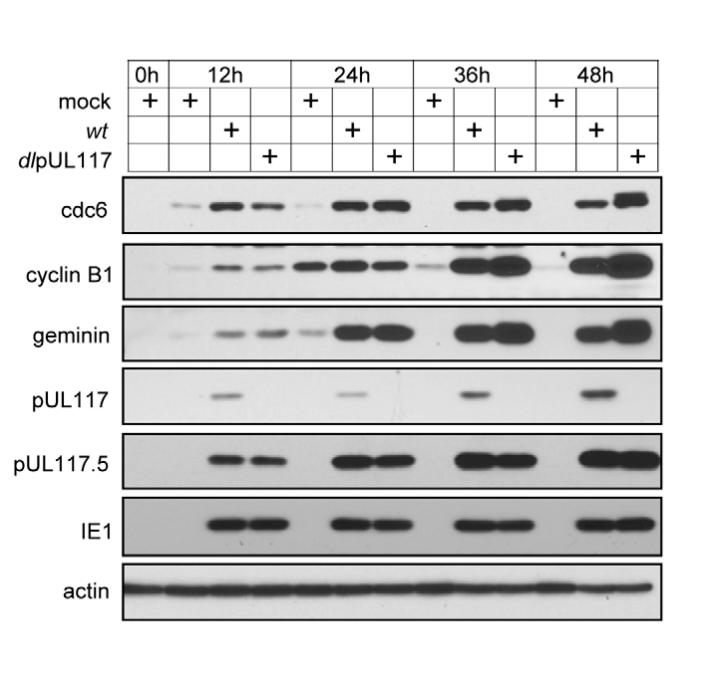
pUL117 was not required for premature accumulation of APC substrates in HCMV infection. Subconfluent HFFs were synchronized at G0 by serum starvation, and then infected with recombinant HCMV in the presence of serum. At indicated times, total lysate from equal number of cells were analyzed by immunoblotting for APC substrates cdc6, cyclin B1, and geminin. Also shown were pUL117-related proteins, IE1 and actin control.

An earlier study suggests that the premature accumulation of geminin, a substrate of the anaphase-promoting complex (APC), may block MCM chromatin loading in HCMV infection [18]. Therefore, we examined the accumulation of geminin and two other APC substrates, cdc6 and cyclin B1, in mutant virus infection. As compared to mock infection, elevated levels of all three APC substrates were evident as early as 12 hpi and persisted throughout the course of infection examined, consistent with previous reports (Fig. 7) [18],[35],[36]. However, this premature accumulation of APC substrates during HCMV infection was independent of pUL117 expression. In fact, these APC substrates accumulated to even greater levels in the absence of pUL117 at 36 and 48 hpi, perhaps due to the increased transcription of these genes at late S and G2 phases in cells infected with the mutant virus. Thus, geminin is unlikely to be targeted by pUL117 to modulate MCM chromatin loading.

Taken together, our results suggest that pUL117 helps to block cellular DNA synthesis during HCMV infection, at least in part, by preventing the accumulation and loading of MCM onto chromatin and subsequent replication licensing.

### pUL117 expression alone is sufficient to inhibit MCM accumulation and reduce cell proliferation

**Figure 8:**
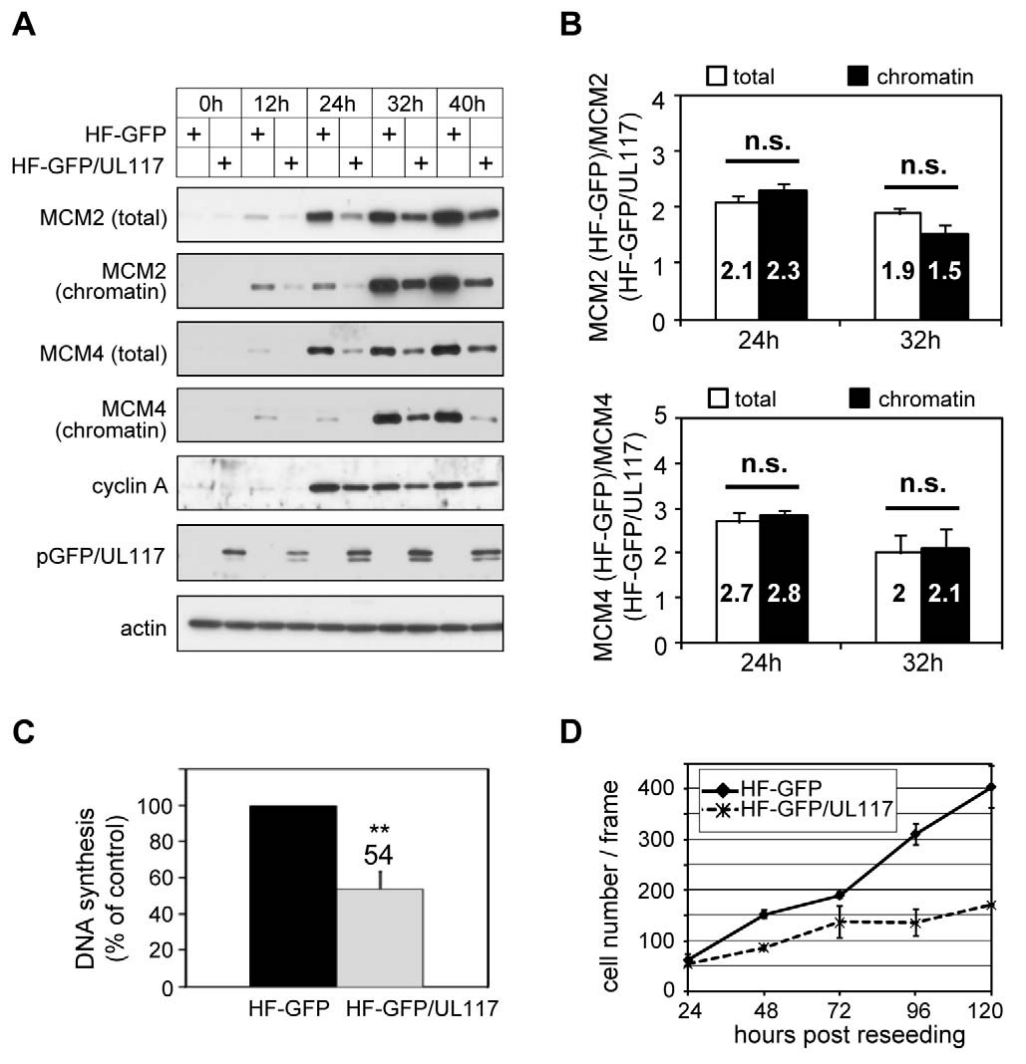
pUL117 was sufficient to inhibit proliferation and cellular DNA replication. HFFs were transduced with retro-viral vector either expressing GFP (HF-GFP) or pUL117 (HF-GFP/UL117) as previously described [20]. (A) Cells were synchronized at G0 by serum starvation, stimulated into the cell cycle by sub-confluent re-seeding with serum, and total cell lysate or chromatin fractions from equal number of cells were examined by immunoblot analysis at indicated times. (B) MCM2 and MCM4 accumulation and their chromatin loading were quantified as described in the legend to Fig. 6B. The ratio of MCM in GFP-transduced cells to that in GFP/UL117-transduced cells was calculated. Shown are the averages of the results from three independent experiments. *, *P*<0.05; **, *P*<0.01; n.s. (not significant), *P*>0.05 by Student's *t* test. (C) At 24 hpi, the rate of DNA synthesis was measured by [^3^H] thymidine incorporation. (D) In a parallel experiment, equal numbers of HF-GFP and HF-GFP/UL117 were re-seeded at sub-confluency with serum and their proliferations were determined by scoring cell numbers during 24–120 h.

We next wanted to test if pUL117 was sufficient to inhibit the accumulation and loading of MCM onto chromatin to block host DNA synthesis. We created HFFs expressing a fully functional GFP-tagged pUL117 (HF-GFP/UL117) [20] or GFP control (HF-GFP) by retroviral transduction. To test the hypothesis, transduced, G0-synchronized cells were stimulated into active growth by re-seeding at sub-confluency with serum, and then analyzed for the rate of DNA synthesis, MCM modulation, and cell proliferation (Fig. 8). In GFP/UL117-expressing cells, the rate of cellular DNA synthesis decreased (54%) compared to GFP-expressing cells (100%) (Fig. 8C). Moreover, the accumulation and chromatin loading of MCM proteins were markedly reduced in these cells (Fig. 8A). Finally, cell proliferation of GFP/UL117-expressing cells was substantially slower relative to GFP-expressing control cells (Fig. 8D). The effect of pGFP/UL117 was unlikely due to non-specific toxic effect resulting from over-expression, since the level of pGFP/UL117 was much lower than that of GFP control in transduced cells (Figure S3). Quantitative immunoblot analysis indicated that both the total and chromatin bound MCMs are indeed markedly reduced in pGFP/UL117 expressing cells, but the magnitude of reduction in the former paralleled that in the latter (Fig. 8B). Thus, pUL117 alone exerts its effect primarily by inhibiting MCM accumulation. During HCMV infection, it may act with other viral factors to exert its additional inhibitory effect to further reduce MCM chromatin loading (Fig. 6B).

### Knockdown of MCM proteins restores the ability of pUL117-deficient virus to block host DNA synthesis

**Figure 9:**
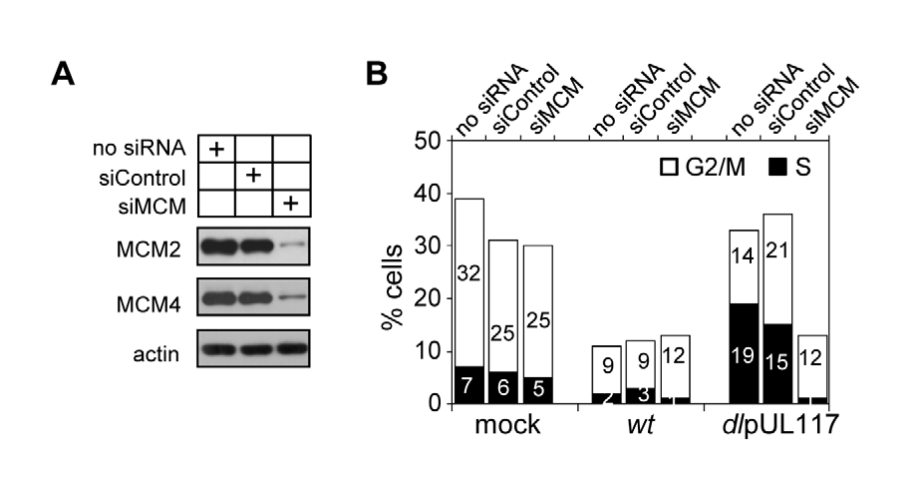
Knockdown of MCM2 and MCM4 restores the ability of pUL117-deficient virus to block host DNA synthesis. HFFs were transfected with a negative control siRNA or siRNAs targeting MCM2 and MCM4. (A) Immunoblot analysis of MCM 2 and MCM4 at 48 hours after siRNA transfection. (B) Transfected cells were starved and infected in the presence of GCV (30µg/ml) as described in Materials and Methods, double-stained with PI and α-pUL44 at 48 hpi, and pUL44+ cells were determined for their DNA content. Percentages of cells in S-phase or G2/M-phases were shown as solid or open bars, respectively. Shown is a representative from three reproducible, independent experiments.

Modulation of MCM function has been suggested as one mechanism for HCMV to block host DNA synthesis [17],[18]. Our study establishes a role for pUL117 in inhibiting MCM function and host DNA synthesis during HCMV infection. We wanted to directly test the role of MCMs in pUL117-mediated inhibition of cellular DNA synthesis. We knocked down MCM2 and MCM4 by siRNA transfection, infect transfected HFFs with HCMV, and examined cellular DNA content in pUL44-positive cells at 48 hpi. MCM-specific siRNAs reduced MCM2 and MCM4 proteins by ∼80% whereas control siRNA only had a negligible effect (Fig. 9A). As expected, wild type virus blocked cellular DNA synthesis regardless of siRNA transfected (Fig. 9B). Also as expected, control siRNA had a minimal effect on the cellular DNA content in pUL117-deficient virus infected cells as 33% of mock- and 36% of control siRNA- transfected cells reached S and G2/M phases (Fig. 9B). In great contrast, transfection of MCM-specific siRNAs restored the ability of pUL117-deficient virus to block cellular DNA synthesis. In mutant virus infection, while 36% of control siRNA-transfected cells reached S and G2/M phases, only 13% of MCM-specific siRNA-transfected cells did so. This inhibitory effect was as potent as that seen during wild type virus infection, in which 13% of cells also reached S and G2/M phases. Thus, targeting MCM function is one key mechanism for pUL117 to block host DNA synthesis.

Together, our results provide evidence that viral protein pUL117 reduces accumulation of MCMs and helps to prevent their loading onto chromatin. This activity is critical for HCMV to block cellular DNA synthesis during infection.

## Discussion

The DNA virus HCMV has an intrinsic ability to stimulate DNA synthesis (Fig. 4), but host DNA synthesis is specifically blocked during infection. Such modulation is pivotal for herpesviruses to establish a successful lytic infection [2]. HCMV proteins pUL69 and IE2 are two known viral proteins that exert this regulatory activity. Inhibition of DNA replication licensing was also proposed as one mechanism for HCMV to block host DNA synthesis [17],[18]. However, the viral factors targeting replication licensing have not been documented. Here, we identified pUL117 as a novel viral factor that inhibits replication licensing by targeting the MCM helicase and blocks cellular DNA synthesis upon HCMV infection. pUL117 expression is sufficient to inhibit MCM protein accumulation and reduce cellular DNA synthesis (Fig. 8). During infection, it may also act with other viral factors to exert its additional inhibitory effect to further reduce MCM chromatin loading (Fig. 6B). Importantly, MCM knockdown by siRNA restores the ability of pUL117-deficient virus to block cellular DNA synthesis (Fig. 9B). Thus, targeting MCM complex is one key mechanism for pUL117 to block host DNA synthesis in HCMV infection. This study also provides evidence to support the previously proposed hypothesis that inhibition of replication licensing is important for HCMV to block cellular DNA synthesis.

It is also noteworthy to mention that other viral factors, such as IE2 [15],[16] and pUL69 [10],[11], contribute to the ability of HCMV to block host DNA synthesis. This is consistent with our results that HCMV was still able to largely prevent host DNA replication at early times and impose additional, post-G1 blocks at late times in the absence of pUL117 (Figs. 2C and 3A). This may also explain why targeting MCM is so efficient to block cellular DNA synthesis in HCMV infected cells but not in uninfected cells. MCMs are normally loaded onto chromatin in a great excess over DNA synthesis initiation events [37],[38]. Knockdown of MCMs by RNAi may not have an immediate effect as minimally licensed chromatin continues to support DNA replication [39],[40]. This is consistent with our result that knockdown of MCM2 and MCM4 only has a subtle effect on the DNA content of uninfected cells (Fig. 9B). However, HCMV encodes several other inhibitory proteins (i.e. IE2 and pUL69) and introduces many changes during infection, which may render cellular DNA synthesis hyper-sensitive to reduced MCM levels. Our results substantiate the general strategy of HCMV to use multiple viral proteins to subvert related cellular processes for its own replication.

As chromatin loading of MCMs precedes the initiation of DNA synthesis at S-phase, it is conceivable that targeting MCM complex by pUL117 enables HCMV to block cellular DNA synthesis while maintaining an S-phase like environment. During infection, several viral proteins, such as pp71, IE1, IE2, and pUL97 inactivate pRb, create a cellular environment conducive to DNA synthesis, and stimulate transcription of E2F-responsive genes, including MCM proteins (reviewed in [5])[1]. On the other hand, accumulation of MCM proteins is delayed and chromatin loading of MCM proteins is reduced in HCMV-infected cells [17],[18]. In addition, HCMV also appears to modulate post-licensing events by blocking hyper-phosphorylation of MCM2 at 24–48 hpi and inducing hyper-phosphorylation of MCM4 at 48–72 hpi in G0-synchronized HFFs [18]. We have also observed MCM4 hyper-phosphorylation at late times during mutant and wild type HCMV infection (data not shown). However, the roles of such modulation need to be further tested. pUL117 appears to target MCM complex primarily by reducing its total levels when expressed alone or during HCMV infection at early times (i.e. 24 hpi). However, the reduction in MCM loading is significantly greater than the reduction in their total accumulation at 30 hpi. Therefore, pUL117 may have other means to target MCM loading in addition to reducing their total accumulation. The exact mechanism for this remains to be elucidated as co-immunoprecipitation studies failed to reveal evidence for a direct interaction of pUL117 with pre-RC components (data not shown). Furthermore, we cannot rule out the possibility that pUL117 may also target other cellular factors to excise its full inhibitory activity.

How does this inhibitory activity of pUL117 impact the replication cycle of HCMV? We previously showed that pUL117-deficient virus was defective in growth and maturation of viral nuclear replication compartments [20]. It is intriguing to speculate that the pUL117-mediated inhibition of host DNA synthesis may prevent downstream detrimental responses to unscheduled cellular DNA synthesis to allow the development of replication compartments, a critical event of the viral infection cycle. Work is in progress to dissect the molecular basis of pUL117 activity and the engagement of MCM in replication compartment maturation during HCMV infection.

DNA viruses employ sophisticated strategies to subvert host DNA replication machinery and cell cycle control pathways to promote their own replication. By driving host cells into an S-phase like environment, viruses promote the accumulation of DNA synthesis components and other cellular factors required for their own production. However, many viruses inhibit host DNA synthesis at critical stages of their replication cycle. Examples include adeno-associated virus (AAV) [41], human papillomavirus (HPV) [42],[43], and herpesviruses [2]. How do these viruses block cellular DNA synthesis in an S-phase like environment? Viruses can accomplish this by modulating an array of DNA replication factors, and MCM proteins emerge as key targets in this strategy. AAV modulates multiple factors required for cellular DNA synthesis. It reduces cyclin-dependent kinase activity and sustains hypophosphorylation of pRb [41],[44],[45]. It also encodes viral DNA replication protein Rep that binds to MCM proteins [46] and inhibits cellular DNA synthesis [44],[45]. It is conceivable that Rep hijacks MCM proteins for viral DNA replication, and simultaneously prevents MCM loading onto host chromatin. Over-expression of the HPV E4 protein prevents MCM protein loading onto host chromatin and inhibits cellular DNA synthesis [42]. Among herpesviruses, Epstein-Barr virus (EBV) induces phosphorylation of MCM4 to inhibit the DNA helicase activity of MCM complex during lytic infections [47]. When over-expressed, an EBV-encoded protein kinase phosphorylates MCM4 and causes cell cycle arrest. However, to our knowledge, the roles of these viral proteins in MCM modulation in the context of virus infection have not been reported. We have identified pUL117 as a viral factor that manipulates MCM during HCMV infection and our results exemplify deregulation of MCM function as a conserved strategy for diverse viruses to prevent host DNA synthesis. Understanding how pUL117 inhibits MCM at a mechanistic level will not only help to elucidate how diversified virus species have evolved to converge on common cellular targets, but may also shed new understanding on how eukaryotic DNA replication is regulated.

## Materials and Methods

### Plasmids and reagents

pYD-C160 and pYD-C305 are pRetro-EBNA based retroviral vectors [48] that express GFP and the GFP-UL117 fusion protein, respectively. Primary antibodies used in this study include: α-pUL117 [20], α-IE1 [49] (a gift from Thomas Shenk at Princeton University), α-pUL44 (Virusys), α-IE1/IE2 (a gift from Jay Nelson at Oregon Health and Science University), α-actin, α-GFP, α-MCM2 (all three from Abcam), α-p16^Ink4a^ (Neomarkers), α-MCM4 (BD Pharmingen), α-lamin A (Biolegend), α-cdc6 (Upstate), α-cyclin B1 (Thermo Scientific), α-cyclin A, α-geminin, α-p53, α-p21 (all four from Santa Cruz Biotechnology). Shld1 was a gift from Thomas Wandless at Stanford University. Ganciclovir (GCV), phosphonoacetic acid (PAA), and propidium iodide (PI) were purchased from Sigma. [Methyl-^14^C] and [Methyl-^3^H] thymidines were purchased from Perkin Elmer. siLentFect lipid reagent was purchased from Bio-Rad. siGENOME Non-Targeting siRNA #2 and siGENOME SMARTpool siRNA targeting human MCM2 and MCM4 were purchased from Thermo SCIENTIFIC. The MCM2 targeted sequences are: 5′-GAA GAU CUU UGC CAG CAU U-3′, 5′-GGA UAA GGC UCG UCA GAU C-3′, 5′-GCC GUG GGC UCC UGU AUG A-3′, and 5′-GGA UGU GAG UCA UGC GGA U-3′. The MCM4 targeted sequences are: 5′-GGA CAU AUC UAU UCU UAC U-3′, 5′-GAU GUU AGU UCA CCA CUG A-3′, 5′-CCA GCU GCC UCA UAC UUU A-3′, and 5′-GAA AGU ACA AGA UCG GUA U-3′.

### Cells and viruses

Primary human foreskin fibroblasts (HFFs) were propagated in Dulbecco's modified Eagle medium (DMEM) supplemented with 10% fetal calf serum. To generate HFFs expressing the GFP-UL117 fusion protein (HF-GFP/UL117) or GFP (HF-GFP), retrovirus stocks were made from transfection of retroviral vector pYD-C305 or pYD-C160, respectively, and then used to transduce HFFs as previously described [50]. Various recombinant HCMV AD169 viruses were reconstituted from transfection of corresponding BAC-HCMV clones as described [20]. Viral stocks were prepared by ultra-centrifuging of infected culture supernatant through 20% D-sorbitol cushion and re-suspending pelleted virus in serum-free medium. pUL117-deficient frame-shift mutant virus AD*dl*pUL117 was created and previously described as AD*in*UL117^C19-*1nt*^ [20]. The recombinant HCMV virus ADpFKBP-UL117 was reconstituted from the BAC-HCMV clone in which UL117 was tagged with a FKBP destabilization domain (ddFKBP) sequence at its 5′-end by linear recombination as described previously [20]. The DNA fragment containing the ddFKBP coding sequence used for recombination was amplified by PCR from plasmid pENTR221-FKBP [27] using a primer pair (5′-CGT GTG GAG CCG TAG ACG ATC TGG ACG TGG TCC TGG GAG AAC ATG ACC ATT TCT TCC GGT TTT AGA AGC T-3′ and 5′-CCC TCA GCC TCT GTG TTC CCA ACA CGT CCC CCC GCT GAG CGT TGG CGG CGA TGG GAG TGC AGG TGG AAA C-3′).

### HCMV infection

To prepare actively growing HFFs, cells synchronized at G0/G1 by contact inhibition were stimulated into the cell cycle by re-seeding at sub-confluency with serum-containing medium for 36 hours, a time where ∼90% of cells were at G1 phase (data not shown). To prepare G0-synchronized HFFs, sub-confluent or confluent cells were starved in serum-free medium for 72 hours. Cells were then infected with recombinant HCMV at a tissue culture infectious dose 50 unit (TCID_50_) of 5, with or without serum (10%) and specific viral DNA replication inhibitors (GCV [30 µg/ml unless indicated otherwise] or phosphonoacetic acid (PAA) [100 µg/ml]) as needed. If GCV or PAA was used, new GCV- or PAA- containing medium was replenished every 48 hours to maintain its efficacy. Where necessary, nocodazole (100 ng/ml) was added at 8 hpi to prevent the second round of the cell cycle progression.

### siRNA knockdown

To knockdown MCM2 and MCM4 in HFFs, subconfluent HFFs were mock-transfected, or transfected with control siRNA or siGENOME SMARTpool siRNA targeting human MCM2 and MCM4 by siLentFect lipid reagent according to manufacturer's instruction (Bio-Rad). Transfected cells were starved in serum-free medium for 3 days and then infected as described above in serum (10%)-containing medium in the presence of GCV (30 µg/ml). Nocodazole (100 ng/ml) was added at 8 hpi to prevent the second round of the cell cycle progression.

### Analysis of cellular and viral DNA

To determine the cellular DNA content, HFFs were removed from culture dish by trypsinization, collected by low-speed centrifugation, fixed, and permeabilized in ice-cold 70% ethanol overnight. Cells were then stained with propidium iodide (PI) only, or double-stained with PI and α-IE1/IE2 or α-pUL44 to identify lytically infected cells. Infected cells that were unsorted, sorted by virus-driven GFP expression, or gated by antibody staining to IE1/IE2 or pUL44 were determined for their DNA content by cell-cycle analysis with flow-cytometry. Percentages of cells in each cell cycle compartment were calculated using the Cell Quest software.

To measure the rate of DNA synthesis, HFFs were first labeled with [^14^C] thymidine overnight. For infection, cells were then starved in serum-free medium for 72 hours before infected with HCMV in the presence of GCV (30 µg/ml). For over-expression, cells were then transduced with expressing retrovirus, starved in serum-free medium for 72 hours, and subsequently stimulated into the cell cycle by sub-confluent re-seeding in serum (10%)-containing medium. At proper times, infected or transduced cells were labeled with [^3^H] thymidine for 2 hours, DNA was recovered by trichloroacetic precipitation, washed with phosphate-buffered saline, and [^3^H] and [^14^C] incorporation were measured by a liquid scintillation counter. The amount of [^3^H] incorporated was normalized to that of [^14^C] incorporated. Experiments were performed in biological triplicate and the *P* value associated with Student's paired *t*-test with a two-tailed distribution was used for statistical analysis.

To prepare intra-cellular DNA for quantification by realtime-quantitative PCR, HCMV-infected HFFs were collected at various times post infection, re-suspended in 2× lysis buffer (200 mM NaCl, 20 mM Tris [pH 8.0], 50 mM EDTA, 0.2 mg/ml proteinase K, 1% sodium dodecyl sulfate [SDS]) that was spiked with 28 ng/ml of a human ATF4 cDNA-containing plasmid [50], and incubated at 55°C overnight. DNA was extracted with phenol-chloroform, precipitated with ethanol, and re-suspended in nuclease-free water (Ambion). Viral DNA was quantified by realtime-quantitative PCR as previously described [20] using a TaqMan probe (Applied Biosystems) and primers specific for the HCMV UL54 gene [12]. Cellular and spiked DNA was quantified with SYBR Advantage qPCR Premix (Clonetech) and primer pairs specific for the human β-actin gene [20] and the human ATF4 cDNA [50], respectively. pUL117-deficient virus and wild type virus differentially modulated cellular DNA synthesis, and therefore the spiked ATF4 cDNA that was in ∼1,000-fold excess to genomic DNA was used to normalize samples. The accumulation of viral DNA was normalized by dividing UL54 gene equivalents by ATF4 cDNA equivalents. The accumulation of pUL117-deficient viral DNA in the presence of GCV was set as 1.

### Protein and transcript analysis

Protein accumulation was analyzed by immunoblotting [20] or flow cytometry. For immunoblotting, cells were collected, washed, and lysed in the sodium dodecyl sulfate (SDS)-containing sample buffer. Proteins from equal cell numbers were resolved by electrophoresis on a SDS-containing polyacrylamide gel, transferred to a PVDF membrane, hybridized with primary antibodies, reacted with HRP-conjugated secondary antibodies, and visualized by SuperSignal West Pico Chemiluminescent Substrate (Thermo Scientific). For quantitative immunoblotting, total cell lysate and chromatin fractions from equal number of cells were diluted in serial two-fold dilutions and then analyzed by immunoblotting for MCM2 and MCM4. The intensity of protein band signals was determined by imageJ software (NIH). Only dilutions that produce protein bands with the intensity within the linear range were used for calculation. To further reduce systematic variations, protein bands from different samples with the most similar intensity were used. The ratio of the MCM in sample A to that in sample B is calculated based on the intensities and dilution factors of the samples: MCM of A / MCM of B = (intensity of A/intensity of B)*(dilution factor of A/dilution factor of B). For flow cytometry, infected HFFs were collected and fixed with 1% paraformaldehyde, labeled with α-pUL44 and Alexa fluor 647 labeled anti-mouse antibody (Invitrogen), and analyzed for expression of pUL44 and HCMV-driven GFP by flow cytometry.

Transcript accumulation was analyzed by reverse transcription-coupled realtime-quantitative PCR as previously described [50]. Total RNA was extracted using the Trizol reagent (Invitrogen) and treated with the TURBO DNA-free reagent (Ambion). cDNA was reverse transcribed with random hexamer primers using the High Capacity cDNA Reverse Transcription Kit (Applied Biosystems), and quantified by real-time qPCR using the SYBR Advantage qPCR Premix (Clontech) and primer pairs specific for human genes MCM4 (5′-TGT GGC AGC ATG CAA AGA A-3′ and 5′-GGG TCA ATA AAA CGC TGA AGA AA-3′) [51] and GAPDH (5′-CTG TTG CTG TAG CCA AAT TCG T-3′ and 5′-ACC CAC TCC TCC ACC TTT GAC-3′). The amount of MCM4 was normalized using GAPDH as the internal control.

### Subnuclear fractionation

Chromatin fractions were prepared to analyze the loading of MCM as previously described [52]. Cells were collected, washed twice with cold phosphate-buffered saline, and lysed in modified CSK buffer (10 mM PIPES, pH 6.8, 100 mM NaCl, 300 mM sucrose, 3 mM MgCl2, 0.5% Triton X-100) containing protease inhibitors (Protease Inhibitor Cocktail, Roche), 1 mM PMSF, phosphatase inhibitors (Phosphatase Inhibitor Cocktail 1 and 2, Sigma), and 1 mM ATP. Cells were incubated on ice for 10 min and then centrifuged at 850×g for 5 min at 4°C. The pellet was washed, re-suspended in lysis buffer by sonication, dissolved in SDS-containing sample buffer, and analyzed by SDS-PAGE and immunoblotting.

## Supporting Information

Figure S1 pUL117 was required to block host DNA synthesis of G0-synchronized HFFs during HCMV infection. Subconfluent HFFs were synchronized at G0 by serum starvation and then infected with recombinant HCMV in the presence of serum. Nocodazole was added at 8 hpi. Cells were infected in the presence phosphonoacetic acid (PAA) (100 µg/ml), double-stained with PI and α-pUL44 at 24 and 48 hpi, and analyzed by flow cytometry for their DNA content and pUL44 expression. Shown is a representative of three reproducible, independent experiments. (A) Signal profiles of pUL44 (x-axis) and PI (y-axis) of mock- or virus- infected cells at 48 hpi. Cells within gate R3 or R4 represents pUL44-negative or pUL44-positive cells, respectively. (B) Mock-infected cells in R3 and virus-infected cells in R4 were analyzed for their DNA content. Percentages of cells in S-phase or G2/M-phases were shown as solid or open bars, respectively.
(7.22 MB TIF)

Figure S2 pUL117 was not required for HCMV to modulate accumulation of p53, p21 or p16 during infection. Subconfluent HFFs were synchronized at G0 by serum starvation, and then infected with recombinant HCMV in the presence of serum. At indicated times, total lysate from equal number of cells were analyzed by immunoblotting for p53, p21 and p16. Also shown were IE1 and actin controls.
(1.79 MB TIF)

Figure S3 The GFP/UL117 fusion protein was not expressed at a higher level than GFP control in stably transduced HFFs. Lysates from equal number of HFFs transduced with retrovirus expressing GFP or pGFP/UL117 were analyzed by immunoblotting with α-GFP antibody.
(1.42 MB TIF)

Figure S4 SSC and FSC gates of HFFs infected with wild type or mutant virus. Shown are the gates for HFFs infected with AD*wt* or AD*dl*UL117 and labeled with α-pUL44 antibody as described in Fig. 2B.
(1.48 MB TIF)

Figure S5 Standard and dissociation curves for MCM4 and GAPDH real time qRT-PCR. Shown are the part of the raw data used to generate the results in Fig. 6C.
(10.05 MB TIF)
