# Supplementary figures and images for: Human Cytomegalovirus Protein pUL117 Targets the Mini-Chromosome Maintenance Complex and Suppresses Cellular DNA Synthesis
Source: PLoS Pathog. 2010 Mar 19;6(3):e1000814. doi: 10.1371/journal.ppat.1000814 (PMC2841624; doi:10.1371/journal.ppat.1000814)

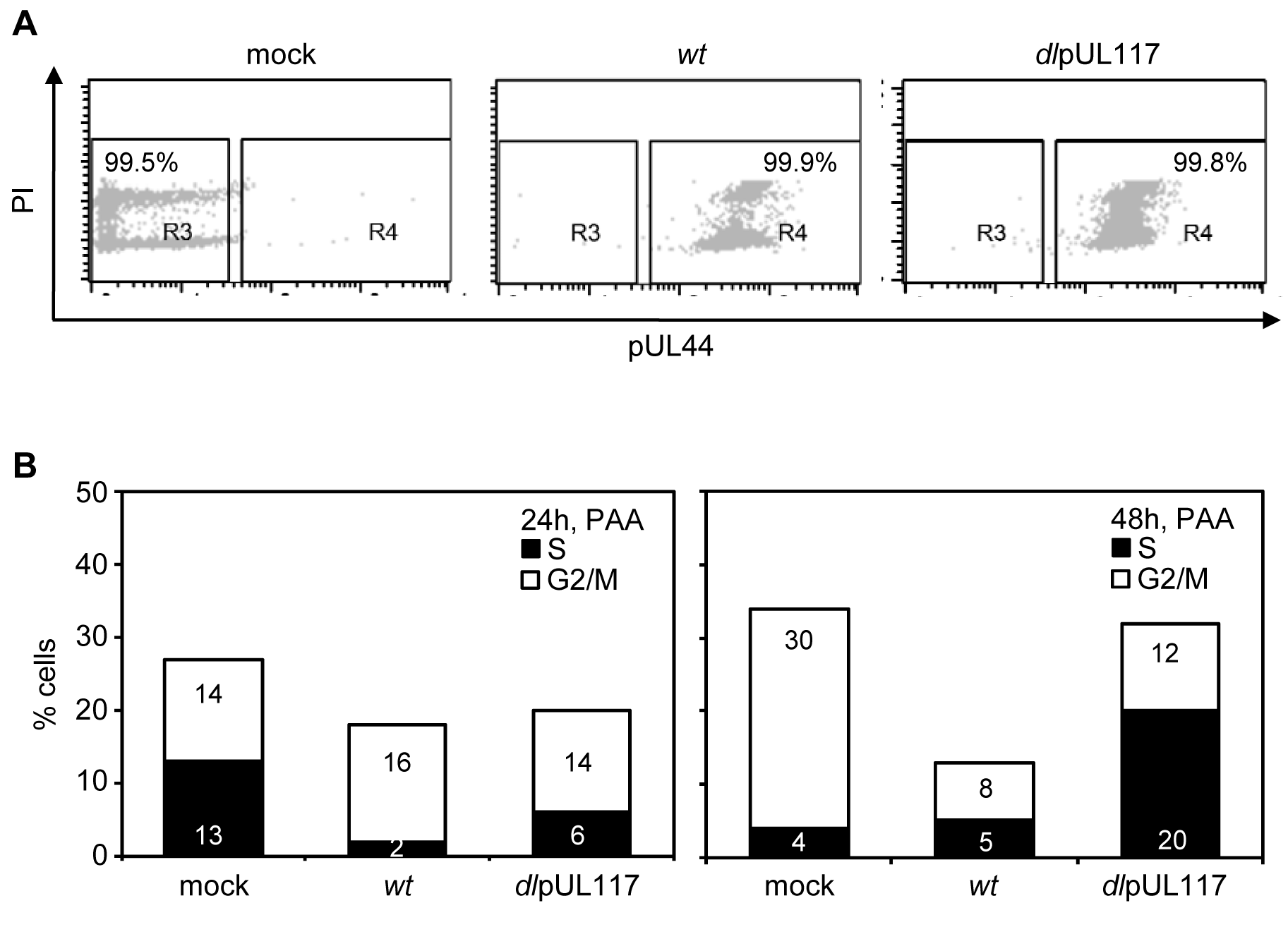

Supplement: Figure S1 — pUL117 was required to block host DNA synthesis of G0-synchronized HFFs during HCMV infection. Subconfluent HFFs were synchronized at G0 by serum starvation and then infected with recombinant HCMV in the presence of serum. Nocodazole was added at 8 hpi. Cells were infected in the presence phosphonoacetic acid (PAA) (100 µg/ml), double-stained with PI and α-pUL44 at 24 and 48 hpi, and analyzed by flow cytometry for their DNA content and pUL44 expression. Shown is a representative of three reproducible, independent experiments. (A) Signal profiles of pUL44 (x-axis) and PI (y-axis) of mock- or virus- infected cells at 48 hpi. Cells within gate R3 or R4 represents pUL44-negative or pUL44-positive cells, respectively. (B) Mock-infected cells in R3 and virus-infected cells in R4 were analyzed for their DNA content. Percentages of cells in S-phase or G2/M-phases were shown as solid or open bars, respectively. (7.22 MB TIF) [file ppat.1000814.s001.tif]

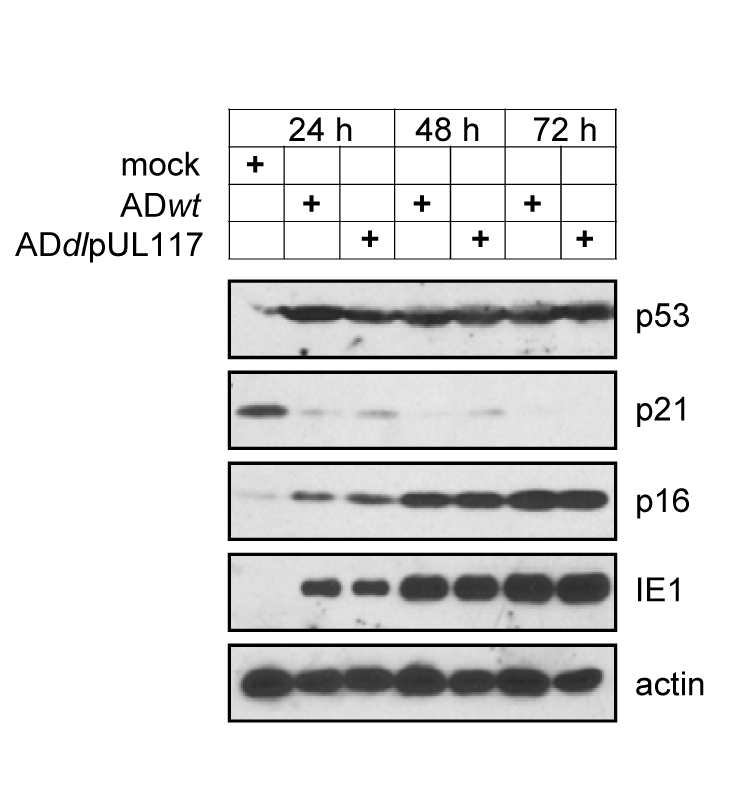

Supplement: Figure S2 — pUL117 was not required for HCMV to modulate accumulation of p53, p21 or p16 during infection. Subconfluent HFFs were synchronized at G0 by serum starvation, and then infected with recombinant HCMV in the presence of serum. At indicated times, total lysate from equal number of cells were analyzed by immunoblotting for p53, p21 and p16. Also shown were IE1 and actin controls. (1.79 MB TIF) [file ppat.1000814.s002.tif]

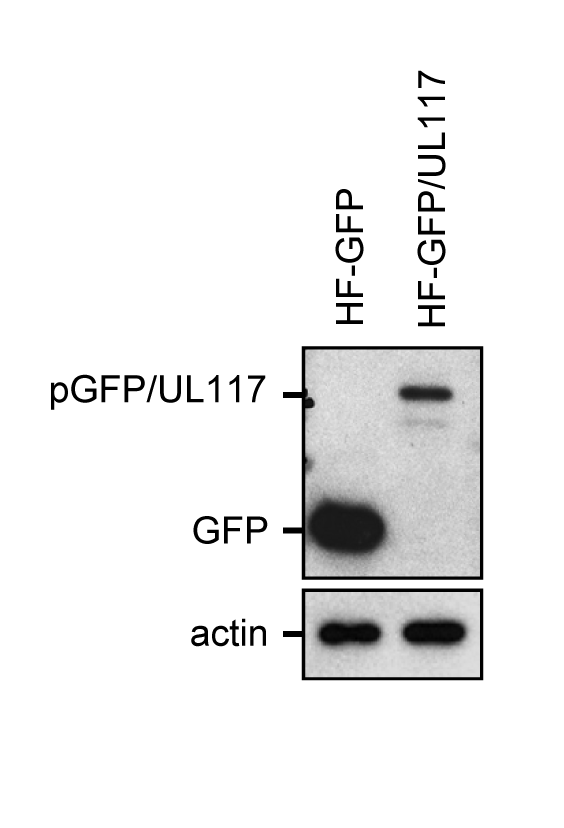

Supplement: Figure S3 — The GFP/UL117 fusion protein was not expressed at a higher level than GFP control in stably transduced HFFs. Lysates from equal number of HFFs transduced with retrovirus expressing GFP or pGFP/UL117 were analyzed by immunoblotting with α-GFP antibody. (1.42 MB TIF) [file ppat.1000814.s003.tif]

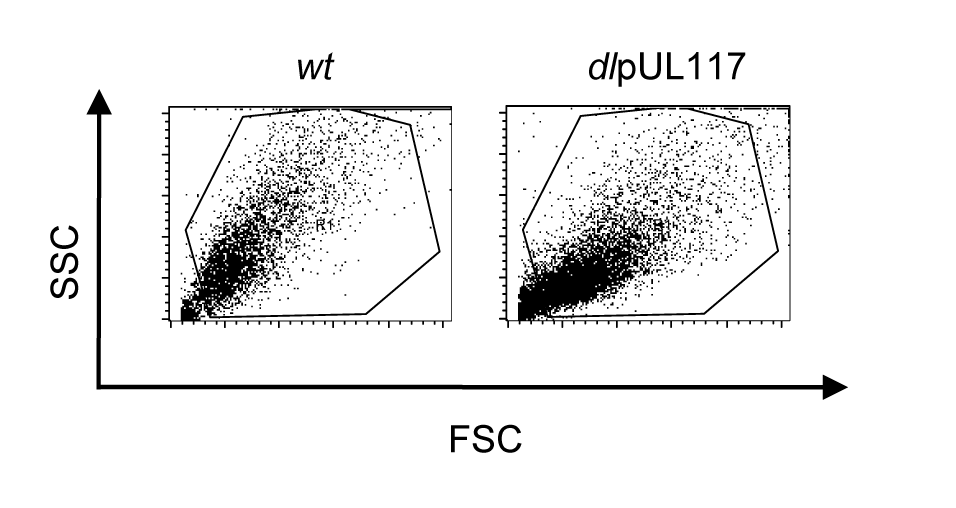

Supplement: Figure S4 — SSC and FSC gates of HFFs infected with wild type or mutant virus. Shown are the gates for HFFs infected with ADwt or ADdlUL117 and labeled with α-pUL44 antibody as described in Fig. 2B. (1.48 MB TIF) [file ppat.1000814.s004.tif]

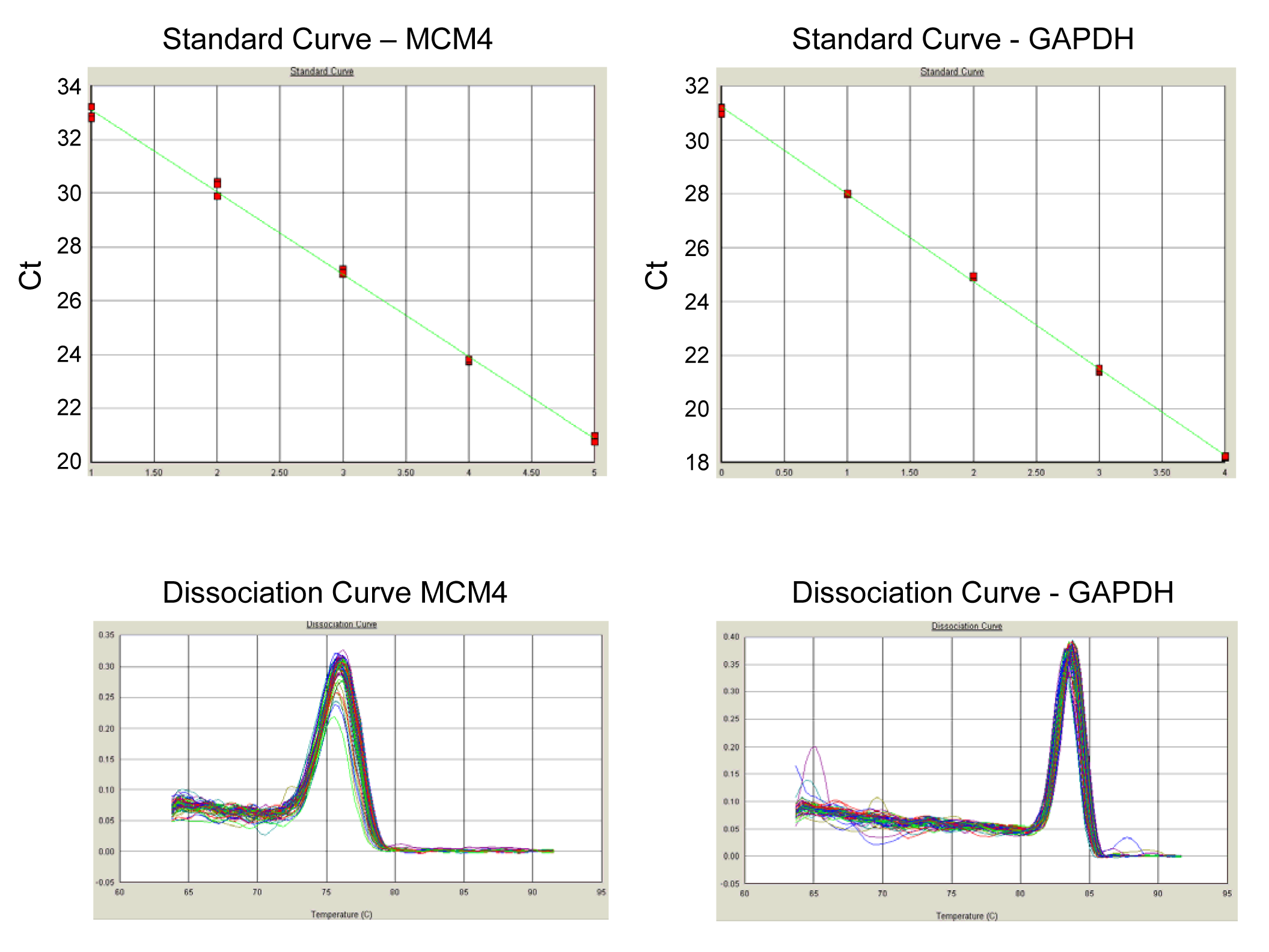

Supplement: Figure S5 — Standard and dissociation curves for MCM4 and GAPDH real time qRT-PCR. Shown are the part of the raw data used to generate the results in Fig. 6C. (10.05 MB TIF) [file ppat.1000814.s005.tif]
